# Early protection events in swine immunized with an experimental live attenuated classical swine fever marker vaccine, FlagT4G

**DOI:** 10.1371/journal.pone.0177433

**Published:** 2017-05-24

**Authors:** Lauren G. Holinka, Vivian O’Donnell, Guillermo R. Risatti, Paul Azzinaro, Jonathan Arzt, Carolina Stenfeldt, Lauro Velazquez-Salinas, Jolene Carlson, Douglas P. Gladue, Manuel V. Borca

**Affiliations:** 1 Plum Island Animal Disease Center, Agricultural Research Service, United States Department of Agriculture, Greenport, New York, United States of America; 2 Department of Pathobiology and Veterinary Science, University of Connecticut, Storrs, Connecticut, United States of America; 3 Oak Ridge Institute for Science and Education, Oak Ridge, Tennessee, United States of America; 4 Biosecurity Research Institute and Department of Diagnostic Medicine and Pathobiology, College of Veterinary Medicine, Kansas State University, Manhattan, Kansas, United States of America; National Institute of Animal Biotechnology, INDIA

## Abstract

Prophylactic vaccination using live attenuated classical swine fever (CSF) vaccines has been a very effective method to control the disease in endemic regions and during outbreaks in previously disease-free areas. These vaccines confer effective protection against the disease at early times post-vaccination although the mechanisms mediating the protection are poorly characterized. Here we present the events occurring after the administration of our in-house developed live attenuated marker vaccine, FlagT4Gv. We previously reported that FlagT4Gv intramuscular (IM) administered conferred effective protection against intranasal challenge with virulent CSFV (BICv) as early as 7 days post-vaccination. Here we report that FlagT4Gv is able to induce protection against disease as early as three days post-vaccination. Immunohistochemical testing of tissues from FlagT4Gv-inoculated animals showed that tonsils were colonized by the vaccine virus by day 3 post-inoculation. There was a complete absence of BICv in tonsils of FlagT4Gv-inoculated animals which had been intranasal (IN) challenged with BICv 3 days after FlagT4Gv infection, confirming that FlagT4Gv inoculation confers sterile immunity. Analysis of systemic levels of 19 different cytokines in vaccinated animals demonstrated an increase of IFN-α three days after FlagT4Gv inoculation compared with mock infected controls.

## Introduction

Classical swine fever virus (CSFV), a member of the genus *Pestivirus* within the family *Flaviviridae*, is the causative agent of Classical swine fever (CSF), a highly contagious disease of swine. CSFV has a positive-sense, single-stranded RNA genome that is contained by an enveloped viral capsid [1].

Control of CSFV is mainly accomplished by two approaches, either prophylactic vaccination in endemic regions or, by “stamping out” of infected and exposed animals in areas free of the disease. Countries free of CSFV do not apply vaccination to their national herds although the currently available CSFV live attenuated viruses (LAVs) confer an effective, rapid and solid immune protection [2]. The reason behind not using these LAVs is the inherent difficulty to differentiate infected and vaccinated animals (i.e., DIVA capability). Therefore, a significant impact on polices regarding CSFV control could be made by the use of a CSFV LAV with DIVA capabilities.

We have previously reported [3,4] the development of a CSFV double antigenic marker LAV strain, with both a positive and negative antigenic markers called FlagT4Gv. This LAV contains an inserted synthetic epitope, Flag^®^ (Sigma, St. Louis, MO), and abolition of a highly conserved CSFV-specific epitope recognized by monoclonal antibody WH303 [5]. Complete protection against challenge with virulent CSFV Brescia was induced by immunization with FlagT4Gv as early as 7 days post-vaccination.

Here we report that FlagT4Gv provides sterile immunity against challenge with the virulent parental virus beginning at day three post-vaccination. In addition, we show that increased levels of IFN-α are present in these animals three days after inoculation with FlagT4Gv.

## Materials and methods

### Viruses, antibodies and cells

BVDV-free swine kidney cells (SK6) [6] were cultured in Dulbecco's Minimal Essential Medium (DMEM) (Gibco, Grand Island, NY) with 10% fetal calf serum (FCS) (Atlas Biologicals, Fort Collins, CO). CSFV BICv and FlagT4Gv were propagated in SK6 cells. Titrations of CSFV from clinical samples were performed using SK6 cells seeded in 96-well plates (Costar, Cambridge, MA). After 4 days in culture, viral infectivity was detected by an immunoperoxidase assay using the CSFV monoclonal antibody (mAb) WH174 (kindly provided by Georgina Ibata, Veterinary Laboratory Agency, UK), mAb WH303[5] or anti-Flag (Sigma, Saint Louis, MO) and the Vectastain ABC kit (Vector Laboratories, Burlingame, CA). Titers were calculated using the method of Reed and Muench [7] and expressed as Tissue Culture Infectious Dose (TCID_50_/mL). As performed, test sensitivity was ≥ 1.8 TCID_50_/mL.

### Animal studies

Protection experiments were performed using commercial breed female pigs weighing 40–60 lbs. Animals were allocated into six groups containing 5 animals each. Animals in five groups were immunized via IM with 10^5^ TCID_50_ of the FlagT4Gv while animals in the remaining group were mock vaccinated. FlagT4Gv inoculated-animals were then intranasal (IN) challenged at either 1, 2, 3, 5 or 7 days post-inoculation (dpi) with 10^5^ TCID_50_ of virulent BICv. Mock treated animals were also IN challenged with 10^5^ TCID_50_ of virulent BICv. Clinical signs and body temperature were recorded daily throughout the experiment as previously described [8]. Viremia was determined using samples collected at 7, 14 and 21 dpi and 4, 7, 11, 15 and 21 days post-challenge (dpc).

For studying early innate immune responses, three animals were IM infected with 10^5^ TCID_50_ of FlagT4Gv and euthanized at 3 dpi, another three animals were IN infected with 10^5^ TCID_50_ of BICv and euthanized 3 days later. A third group of three animals was IM infected with 10^5^ TCID_50_ of FlagT4Gv, IN challenged 3 days later with 10^5^ TCID_50_ BICv, and euthanized 3 days after the challenge. An additional two animals were used as naive controls (receiving no virus). Tonsils and blood samples were collected after euthanasia from all the 11 swine.

### Ethics statement

Animal experiments were conducted using research protocols approved by the Plum Island Animal Disease Center IACUC committee (Protocol Number: 171.02-15-R: Classical swine fever virus (CSFV): evaluation of virulence of wild type and genetically modified viruses). The animals were obtained from Animal Biotech Industries (Danboro, PA). It should be noted that animals developing CSFV disease signs were euthanized as soon as they fit the criteria described in the corresponding IACUC protocol.

### Immunofluorescence and confocal microscopy

Triplicate samples were collected postmortem from the tonsils. The tissues were mounted on blocks using optimal cutting temperature (OCT) compound (TissueTek, Sakura Finetek USA, Torrance, CA) and promptly frozen in liquid nitrogen, then stored at -70°C. Five μm thick sections of cryopreserved tissues from all three triplicate specimens from each tissue of all three animals were sectioned with a cryomicrotome and fixed with acetone for 10 min at –20°C. After fixation, the sections were incubated in blocking buffer (Phosphate Buffered Saline, containing 0.05%Tween-20 [Sigma, Saint Louis, MO], 6% normal bovine serum, 6% normal goat serum, 2% skim milk) for at least 4 hours at room temperature (RT). Primary antibodies were diluted in blocking buffer and incubated with the slides overnight at 4°C in a humidified chamber. When double labeling was performed, the slides were incubated with both the antibodies together. The dilutions of the primary antibodies were as follows: monoclonal antibody WH303 (1/100), anti-Flag M2 monoclonal antibody (1/50; Sigma,), rabbit anti-pan cytokeratin (1/100; Invitrogen, Carlsbad, CA), monoclonal antibody anti-monocyte-granulocyte (1/100; BDPharmigen, San jose, CA). After washing five times with PBS-Tween, the slides were incubated with the appropriate secondary antibodies, goat anti-mouse isotype-specific IgG (1/400, AlexaFluor 488 or AlexaFluor 594, Molecular Probes [Eugene, OR]) diluted in blocking buffer for 0.5 hours at 37°C. Following this incubation, the slides were washed 5 times with PBS-Tween, counterstained with the nuclear staining TOPRO-iodide 642/661 (Molecular Probes) for 5 minutes at RT, washed as before, mounted with ProLong antifade reagent (Thermo Fisher, Waltham, MA), and examined with a Zeiss LSM 710 confocal microscope. Data were collected utilizing an appropriate control lacking the primary antibodies in order to determine channel crossover settings, as well as using WH303 and anti-Flag monoclonal antibodies in uninfected tissues to give the negative background level and to determine channel cross-over settings. The captured images were adjusted for contrast and brightness using Adobe Photoshop® software.

### Detection of cytokines in sera of FlagT4Gv-infected animals

Levels of serum MCP2 (monocyte chemoattractant protein 2), TGF-β1 (transforming growth factor beta 1), IFN-α (interferon alpha), IFN-β (interferon beta), IFN-γ (interferon gamma), IL-1α (interleukin 1 alpha), IL-1β (interleukin 1 beta), IL-2 (interleukin 2), IL-5 (interleukin 5), IL-8 (interleukin 8), IL-10 (interleukin 10), IL-12 p35 (interleukin 12 p35), IL-12 p40 (interleukin 12 p40), OAS (oligoadenylate synthetase), PKR (protein kinase R), TNF (tumor necrosis factor), MX-1 (mixovirus resistance), and VCAM (vascular cell adhesion molecule 1)were assessed using commercial ELISAs following manufacturer protocols (MyBioSource, San Diego, CA) as previously described [9].

## Results and discussion

### Assessment of early protection induced by FlagT4Gv

**Fig 1:**
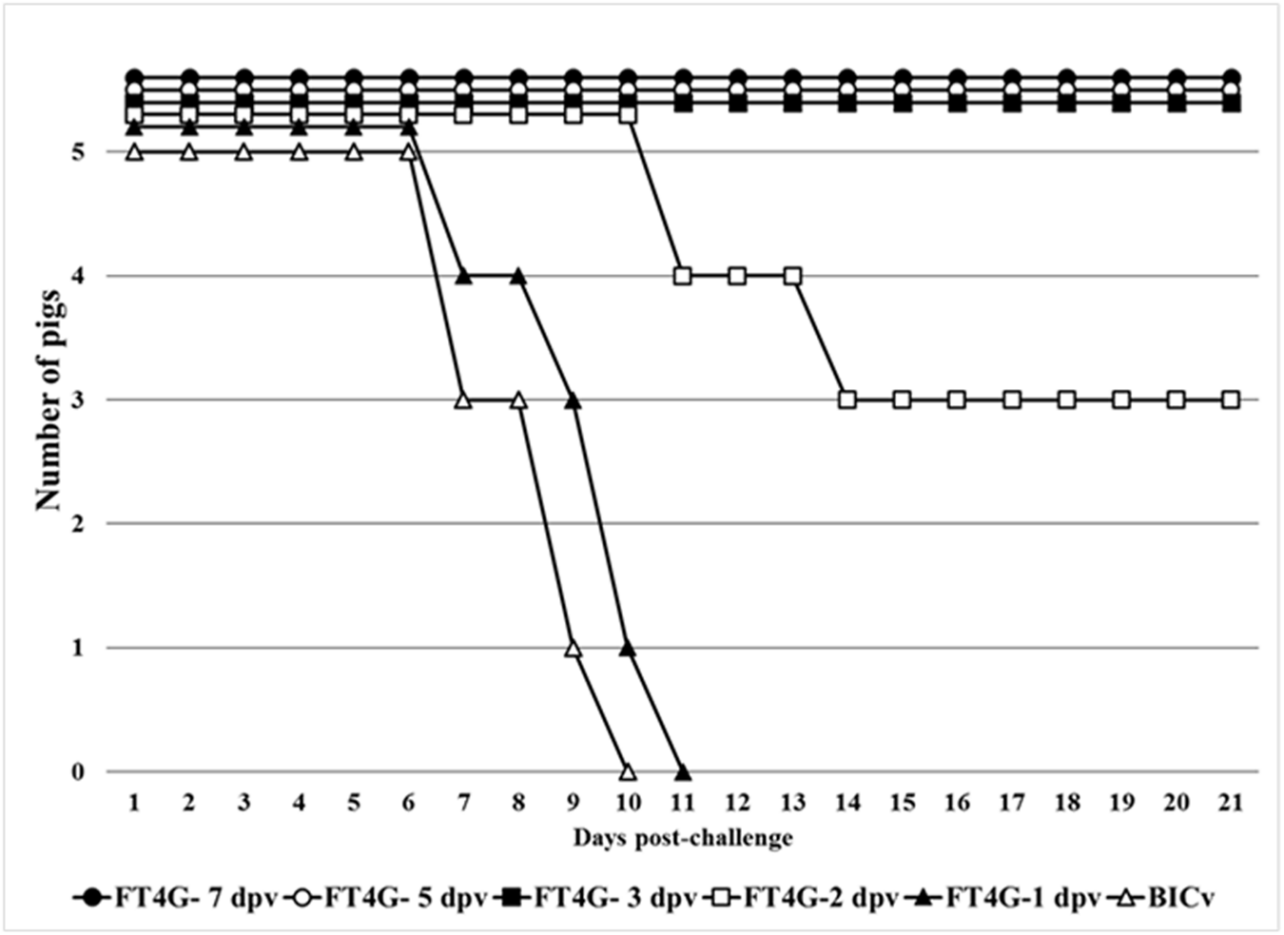
Mortality in FlagT4Gv-infected animals challenged at different times post-infection with virulent BICv. Groups of animals (n = 5) were IM inoculated with 10^5^ TCID_50_ of FlagT4Gv and IN challenged with BICv 1, 2, 3, 5 or 7 days later. Animals were clinically observed for 21 days post-challenge.

FlagT4G virus (FlagT4Gv), like other CSFV LAV strains, is able to induce immunity within the first week post-vaccination. We previously reported that 7 days after being inoculated with FlagT4Gv, animals were completely protected against disease when challenged with virulent parental BICv [4]. Consequently, we investigated how early after FlagT4Gv inoculation the animals could be protected against clinical disease presentation and/or infection when challenged with BICv. Five groups of animals (n = 5) were IM inoculated with 10^5^ TCID_50_ of FlagT4Gv and were challenged by the IN administration of 10^5^ TCID_50_ of BICv 1, 2, 3, 5, or 7 days later. Protection was evaluated by daily clinical monitoring of the animals (including the recording of body temperature) during a 21 day observation period. Mock-vaccinated and challenged animals presented with typical CSF disease starting at 5 dpc, with all animals euthanized by 8–10 dpc (Fig 1 and Table 1). All animals challenged on the first day after inoculation with FlagT4Gv presented disease kinetics similar to that of the mock treated animals (Table 1 and Fig 1). Animals challenged on the second day after inoculation with FlagT4Gv presented an intermediate outcome. Two of the five pigs developed a severe form of CSF starting at 6–7 dpc with a disease progression similar to that of the mock treated animals, and were euthanized by 11–14 dpc. One of the five pigs had a transient fever without the presence of additional clinical signs while the other two animals in the group remained completely asymptomatic (Table 1 and Fig 1). Importantly, animals in groups challenged at day 3, 5 or 7 after FlagT4Gv infection remained completely asymptomatic throughout the observation period (Table 1 and Fig 1).

**Table 1:** Swine survival and fever response in FlagT4Gv-infected animals challenged with BICv at different times post-infection.

			Fever		
FlagT4Gv / BICv ^(1)^ challenged performed at	No. of survivors/total	Mean time to death (days ± SD)	No. of days to onset (days ± SD	Duration No. of days (days ± SD)	Maximum daily temp (°F ± SD)
Mock ^(2)^	0/5	8.4 (1.34)	5 (0)	3.4 (1.34)	105.6 (0.42)
FlagT4Gv / 1 dpi	0/5	9.4 (1.52)	5 (0)	4 (1.52)	106.4 (1.22)
FlagT4Gv / 2 dpi	3/5	12.5 (2.12) ^[^^10^^]^	6.8 (1.26) ^[^^10^^]^	4.5 (2.36) ^[^^10^^]^	105.5 (1.66) ^(3)^
FlagT4Gv / 3 dpi	5/5	-	-	-	103.4 (0.58)
FlagT4Gv / 5 dpi	5/5	-	-	-	103.4 (0.65)
FlagT4Gv / 7 dpi	5/5	-	-	-	103.4 (0.31)

(1) FlagT4Gv was inoculated IM at a dose of 10^5^ TCID_50_ while BICv was inoculated IN at a dose of 10^5^ TCID_50_.

(2) Mock treated animals were also challenged with BICv.

[10] Data are based on 2 of 5 animals presenting severe disease symptoms and ultimately euthanized at days 11 and 14 post-challenge, and 1 of 5 animals presenting transitory rise of body temperature without any other CSF-related symptom. The other two animals remained clinically normal with maximum average daily body temperature of 103.6°F.

**Fig 2:**
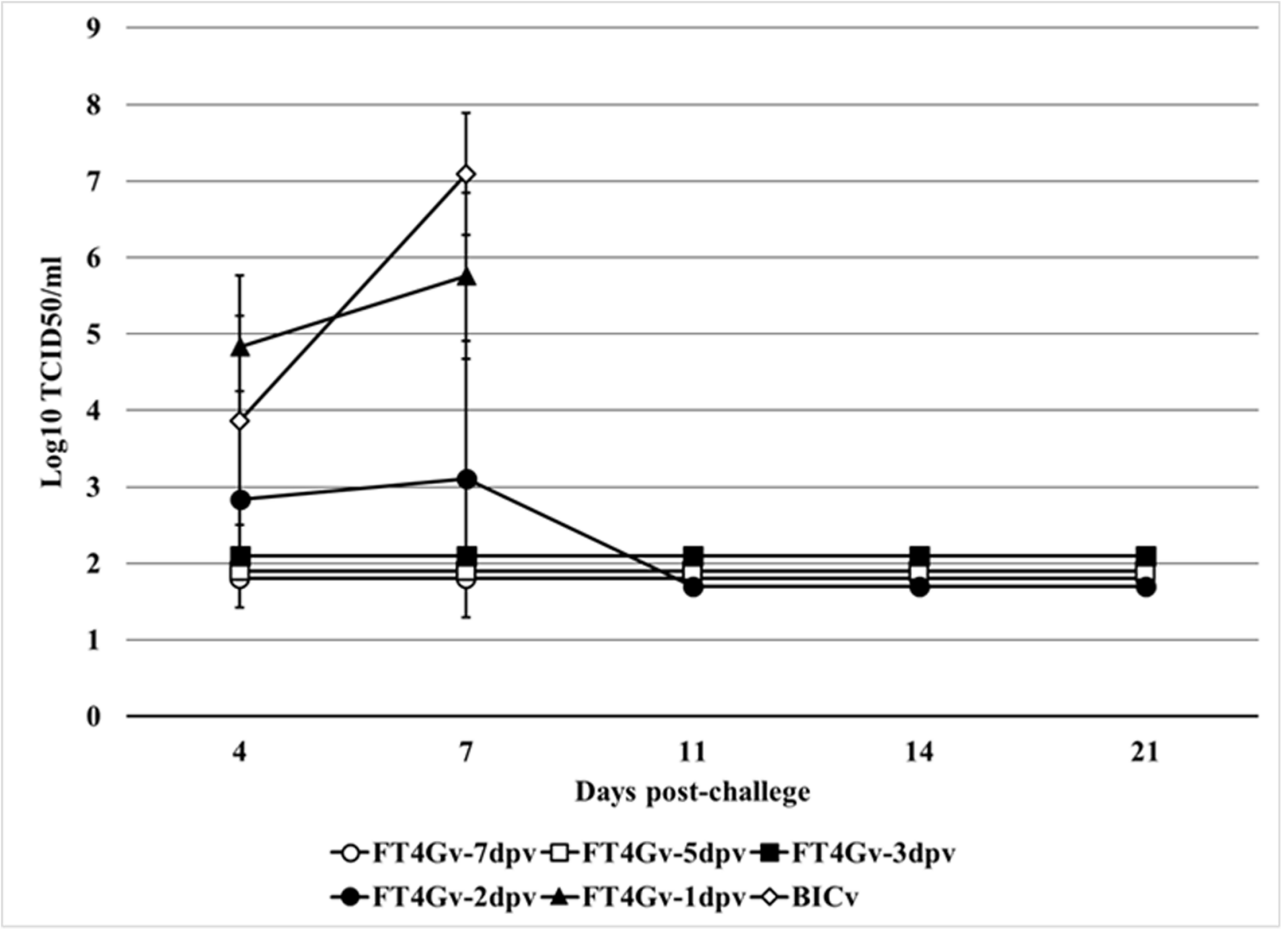
Viremia in FlagT4Gv-infected animals challenged at different times post-infection with virulent BICv. Data represent average titers and SD of 5 animals in each time point. Titers, expressed as TCID_50_/mL, correspond exclusively to presence of BICv that was determined by immunocytochemistry using mAbs WH303 which specifically detects BICv. Sensitivity of virus detection: ≥1.8 TCID_50_/mL.

Viremia in BICv-challenged animals followed the appearance of clinical signs and virus titers correlated with severity of the observed clinical disease (Fig 2). Mock treated animals presented typical high titer values at 4 and 7 dpc. Animals challenged at 1 dpi also presented high titer values at 4 and 7 dpc. Average titers were significantly lower in the group of animals challenged at 2 dpi due to the fact that surviving animals did not present detectable viremia. Viremia in animals challenged at 3, 5 or 7 dpi was consistently undetectable throughout the observation period. These results indicate that beginning at 3 dpi, animals infected with FlagT4Gv became solidly protected against challenge with the virulent parental virus in terms of both the appearance of clinical signs associated with the disease and replication of the challenge virus. Based on the *in vivo* challenge studies and *in vitro* virus titer experiments, we conclude that FlagT4Gv provides efficient protection against CSF disease as early as 3 days post-inoculation.

The usefulness of an emergency vaccine depends on how early the vaccine provides protection against lethal infection. The CSFV C strain, the “Gold Standard” for CSF vaccination, has been characterized to induce protection rapidly. As previously reviewed [2], pigs vaccinated with the C strain are partially protected within 2–4 days of vaccination [11,12] and are completely protected within 5 to 7 days [6,12–18], with sterile immunity achieved by 7 days post-vaccination [14,15,19,20]. Research performed with the live attenuated marker vaccine candidate Cp7_E2alf (harboring the E2 gene of CSFV strain Alfort in a bovine viral diarrhea virus genetic backbone) was also shown to protect animals against virulent CSFV strain Koslov as early as 7 days post-vaccination when IM administered and 14 days post-vaccination when delivered orally [21]. In both cases, all vaccinated animals were completely protected against lethal CSFV challenge. Animals IN vaccinated with either CP7_E2alf vaccine or C strain and challenged with moderately virulent CSFV isolate Bas-Rhin at 2 days post-vaccination were only partially protected against development of disease [22]. In this report, we achieved rapid (3 days post-vaccination), complete (sterile immunity) protection induced by FlagT4Gv against experimental challenge with a highly virulent strain of CSFV.

### Comparative growth of FlagT4Gv and BICv in swine macrophages

**Fig 3:**
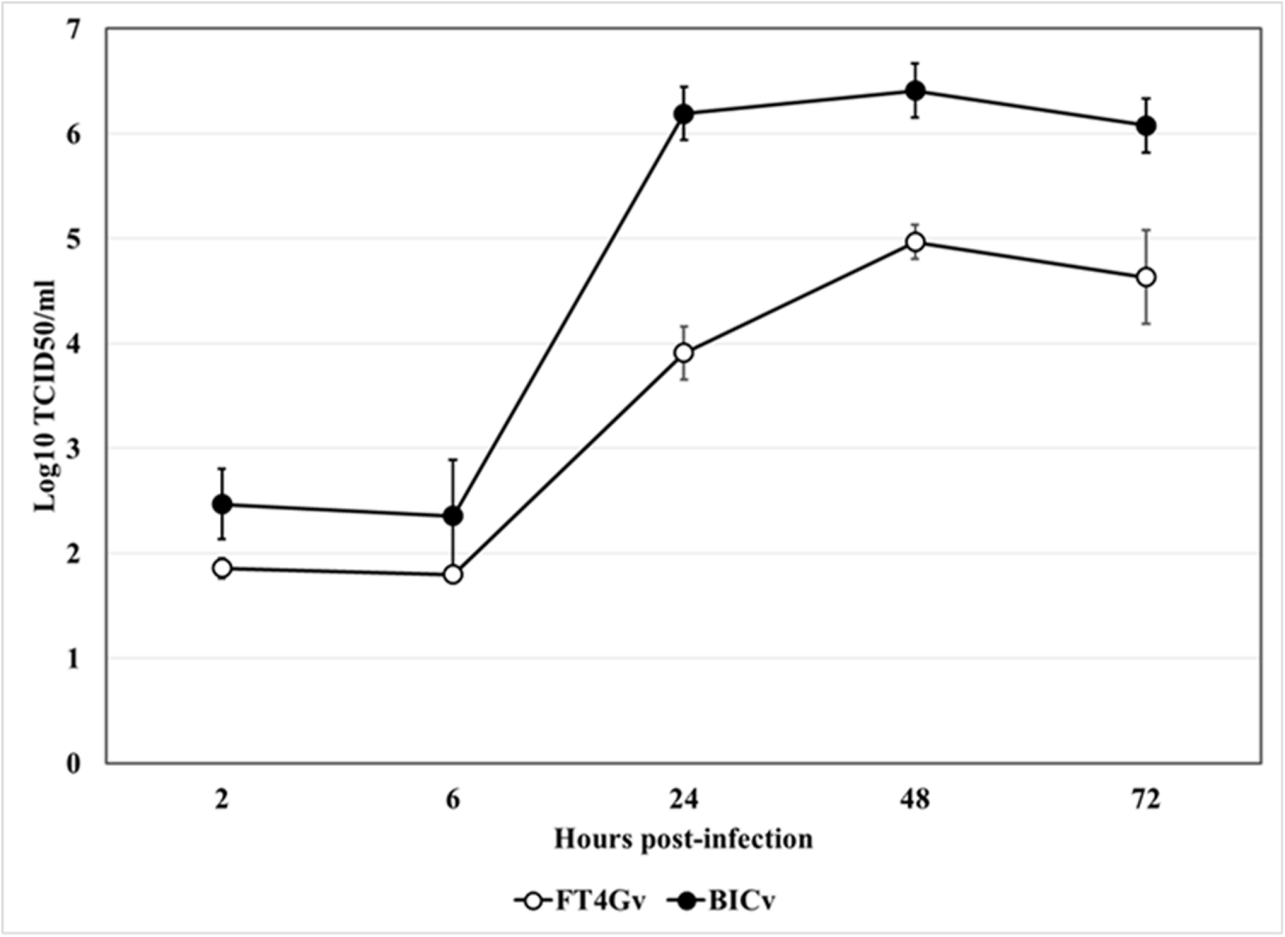
*In vitro* growth of FlagT4G virus. Primary cell cultures of swine macrophages were infected (MOI = 0.01) with FlagT4G or BIC viruses and virus yield titrated at the indicated times post-infection in SK6 cells. Data represent means and standard deviations from two independent experiments. Sensitivity of virus detection: ≥1.8 TCID_50_/mL.

The ability of FlagT4Gv to replicate in swine macrophages, the primary cell targeted by CSFV during infection in swine, was evaluated and compared relative to parental BICv in a multistep growth curve using primary swine macrophage cell cultures (Fig 3). Macrophage cell cultures were infected at a MOI of 0.01 and samples were collected at 2, 6, 24, 48, and 72 hours post-infection (hpi). FlagT4Gv displayed a growth kinetic significantly lower than parental BICv. Depending on the time-point considered, FlagT4Gv exhibited titers 10- to 100-fold slower relative to the parental virus. Therefore, FlagT4Gv’s ability to replicate in swine macrophages is significantly diminished when compared to its parental virus.

### Virological and immunological events three days after FlagT4Gv infection

**Fig 4:**
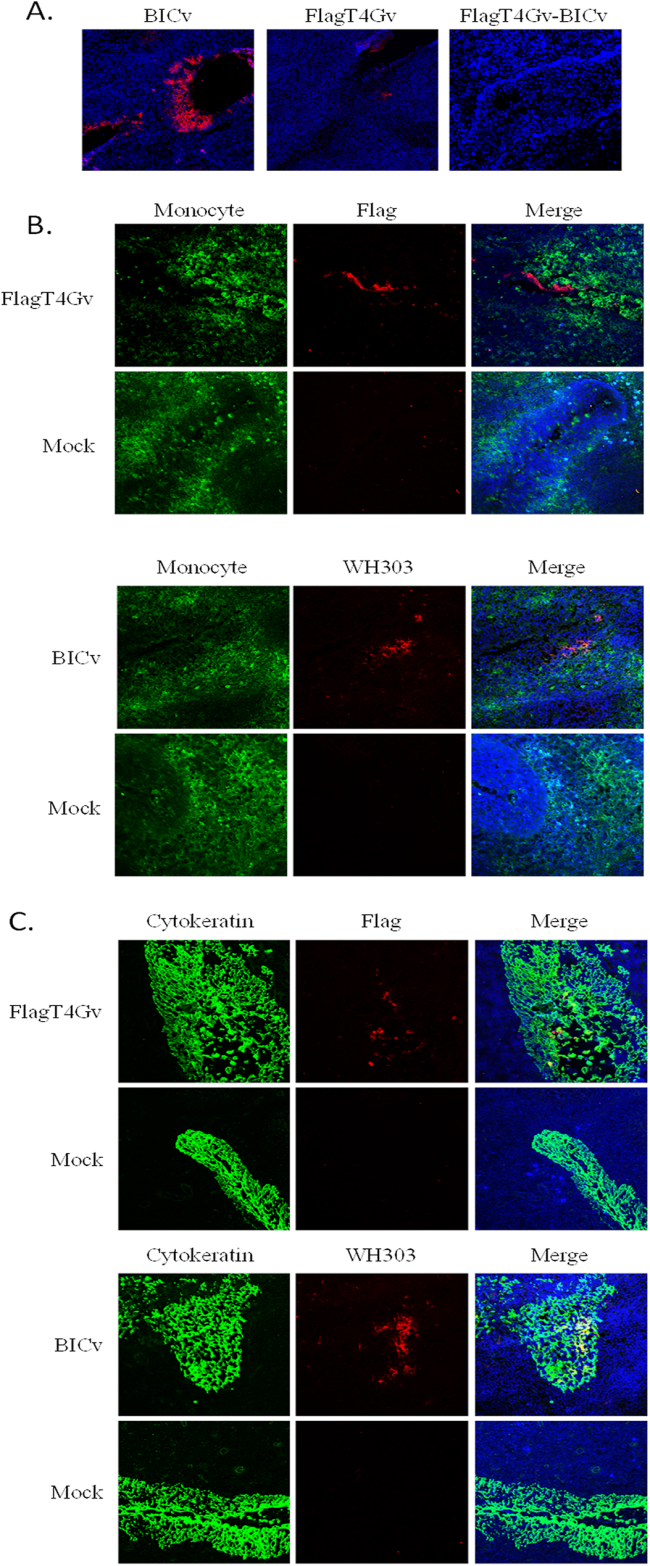
Localization of BICv and FlagT4Gv in tissue samples. **(A)** Localization of BICv and FlagT4Gv in frozen sections of tonsil tissues from pigs either IM infected with FlagT4Gv or IN with BICv. Cryosectioned tissues from the tonsils were processed for immunofluorescence and confocal microscopy using monoclonal antibodies WH303 (specific for BICv) or anti-Flag (Specific for FlagT4Gv), respectively. The virus was visualized with Alexa Fluor 563 (red). Sections were counterstained with TOPRO-iodide 642/661 (blue) to reveal the nuclei. **(B)** Co-expression of CSFV E2 303 epitope (specific for BICv) or Flag epitope (specific for FlagT4v) along with Monocyte/Graculocyte cell marker in tonsil sections of FlagT4Gv or BICv infected pigs. Cryosectioned tissues were labeled for immunofluorescence and confocal microscopy with monoclonal antibody WH303 or anti-Flag monoclonal antibody along with anti-Monocyte/Granulocyte marker antibodies. Viral antigens were visualized with Alexa Fluor 563 (red), Monocyte /Granulocyte was visualized with Alexa Fluor 488 (green). Sections were counterstained with TOPRO 642/661 (blue) to reveal the nuclei. **(C)** Co-expression of CSFV E2 303 epitope (specific for BICv) or Flag epitope (specific for FlagT4v) along with cytokeratin in tonsil sections of FlagT4Gv or BICv infected pigs. Cryosectioned tissues were labeled for immunofluorescence and confocal microscopy with monoclonal antibody WH303 or anti-Flag monoclonal antibody along with anti-cytokeratin antibodies. Viral antigens were visualized with Alexa Fluor 563 (red), and Cytokeratin was visualized with Alexa Fluor 488 (green). Sections were counterstained with TOPRO 642/661 (blue) to reveal the nuclei.

At 3 dpi, we evaluated the immune response in animals infected with FlagT4Gv, as well as FlagT4Gv-infected animals that were challenged with BICv, and compared those with animals infected only with virulent BICv. Three groups composed of 3 pigs each were treated as follows: (i) IM inoculation with 10^5^ TCID_50_ of FlagT4Gv and euthanized 3 days later, (ii) IN inoculation with 10^5^ TCID_50_ of BICv and euthanized 3 days later, and (iii) IM inoculation with 10^5^ TCID_50_ of FlagT4Gv followed by an IN challenge with 10^5^ TCID_50_ BICv 3 days later, and then euthanized at the third day post challenge (DPC) (Table 2). An additional two animals were used as naive uninfected controls. Tonsils were collected after euthanasia from all the 11 swine. Blood samples were also collected at the time of death. Detection of both viruses in tonsils was performed by virus titration in SK6 cell cultures and using specific monoclonal antibodies (anti-Flag for FlagT4Gv and WH303 for BICv). High titers of BICv (4.63 log_10_ TCID_50_/mL, ±SD:0.00) were detected in tonsils of all three animals IN infected with BICv three days earlier (group ii), indicating that these animals suffered a massive infection with the challenge virus. On the other hand, tonsils from animals IM inoculated three days earlier with FlagT4Gv (group i) had almost undetectable titers (2.02 log_10_TCID_50_/mL, ±SD:0.09), suggesting that the tonsils were barely colonized by FlagT4Gv at this time post-infection. Interestingly, BICv cannot be detected (sensitivity of virus detection: ≥1.8 TCID_50_/mL) in tonsils of FlagT4Gv-infected animals three days after challenge with BICv (group iii). These results indicate that infection with FlagT4Gv completely prevents the replication of BICv in tonsils. Confirmation of these results was obtained by analyzing the tonsils of these animals by immunohistochemistry using the monoclonal antibodies against Flag and WH303 epitopes. Correlating with virus titers, abundant large WH303-positive foci were detected in animals three days after they were IN infected with BICv (group ii) (Fig 4A). Conversely, few and small Flag-positive foci were detected in tonsils of animals that were IM infected with FlagT4Gv three days earlier (group i) (Fig 4A). Importantly, no WH303 antibody reactivity could be detected in tonsils of FlagT4Gv-inoculated and BICv-challenged animals (group iii) (Fig 4A). No nonspecific fluorescent activity was detected in tonsils of mock infected animals when evaluated using Flag and WH303 monoclonal antibodies (Fig 4B and 4C).

**Table 2:** Description of the second animal experiment.

	Action performed at Day		
Treatment ^(1)^	0	3	6
Mock	None	None	Euthanasia
(i) FlagT4Gv	FlagT4Gv inoculation	Euthanasia	None
(ii) BICv	BICv inoculation	Euthanasia	None
(iii) FlagT4Gv + BICv	FlagT4Gv inoculation	BICv inoculation	Euthanasia

(1) FlagT4Gv was inoculated IM at a dose of 10^5^ TCID_50_ while BICv was inoculated IN at a dose of 10^5^ TCID_50_.

WH303 reactivity was strongly correlated with epithelial crypts in BICv-infected animals, while most of the Flag reactivity was associated with a few individual cells within epithelial crypts. Further characterization of antigen-positive cells was performed utilizing dual labeling, with antibodies against cytokeratin, an epithelial marker, and to a macrophage-granulocyte marker. In general, cells positive for monoclonal antibodies WH303 and Flag also expressed cytokeratin while no cells were positive for a macrophage-granulocyte marker expression (Fig 4B and 4C).

Results indicate that after IM inoculation a very small quantity of FlagT4Gv colonizes tonsils while IN-inoculated BICv is completely unable to establish infection in tonsils of animals which were previously infected with FlagT4Gv. In addition, both viruses appears to exclusively infect epithelial cells of the tonsil.

As discussed earlier, protection induced in C-strain vaccinated animals at 5 to 7 dpv appears to also confer sterile immunity when challenged [6,13–20]. FlagT4Gv is the first reported CSFV vaccine candidate to induce sterile protection against a highly virulent CSF virus as early as three days post-inoculation.

As expected, all sera from the 11 animals included in this experiment were negative for CSFV-specific antibodies detected using a commercially available ELISA (data not shown) (PrioCHECK, Thermo-Fisher, Waltham, MA). As reviewed previously [2] during the first week after vaccination no evidence of a virus-specific immune response is detected. This suggests that mechanisms mediating early protection may belong to the innate immune response.

**Fig 5:**
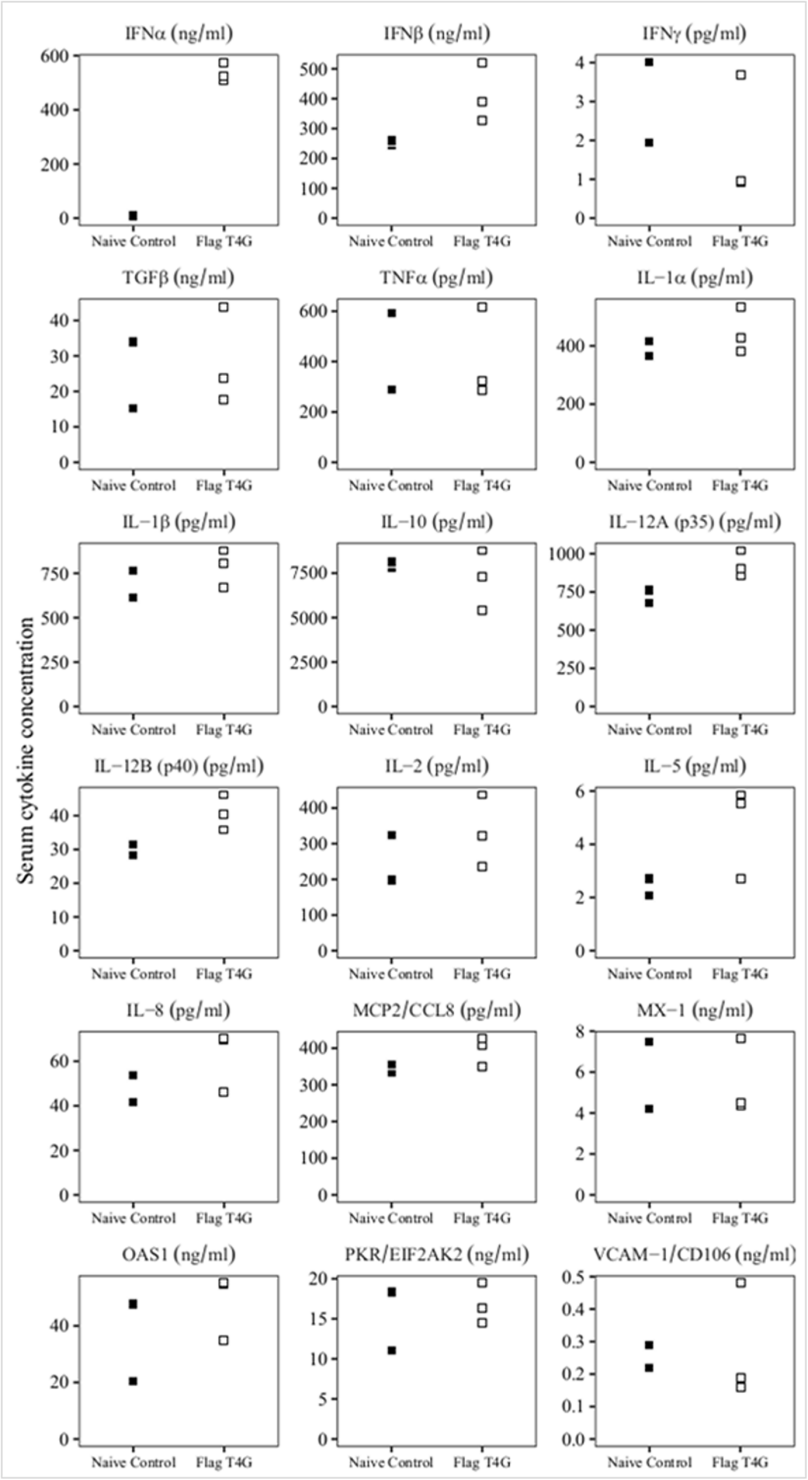
Evaluation of the systemic levels of different host cytokines in swine. Samples were taken on the third day after inoculation with FlagT4G virus (FlagT4Gv), or mock infection (naïve control). Data represent individual animal determinations. Values are expressed as concentration per mL of serum determined as described in Material and Methods.

Therefore, it was important to evaluate the possible role of innate immunity in protection at early time points after FlagT4Gv inoculation. For that purpose, the levels of multiple cytokines present in serum of animals at the third day after they were IM inoculated with FlagT4Gv (group i, in the previous experiment) were assessed by ELISA and compared with those in the mock treated animals. Specifically, levels of MCP2, TGF-β1, IFN- α, IFN-β, IFN-γ, IL-1α IL-1β, IL-2, IL-5, IL-6, IL-8, IL-10, IL12-p35, IL12-p40, OAS, PKR, TNF, MX-1, and VCAM were assessed using commercial ELISAs following manufacturer protocols (MyBioSource). The results of the ELISAs were used to try to establish an association between mediators of the innate host immune response and infection with FlagT4Gv (Fig 5). The average cytokine values did not significantly vary between the FlagT4Gv infected pigs and mock treated animals. Remarkably, significant differences were only observed regarding IFN-α levels between groups. At three days post-FlagT4Gv-infection, circulating IFN-α was determined to have a concentration of 536.72 ng/mL (SD±36.26). This concentration was much higher than 8.19 ng/mL (SD±1.34) of mock infected animals. These results suggest that IFN-α may play a significant role as a mediator of innate immune response against BICv challenge.

Although the ability of the host innate immune system to interact with CSFV replication has been studied *in vitro* by several groups [23–27] the innate response factors mediating protection at early times post-vaccination remain unknown. Notably, changes in the expression of IFNs due to CSFV infection have been reported previously [26–28]. Previous reports have shown that CSFV possesses mechanisms that hinder the induction and production of IFNs [27,28]. Furthermore, a direct correlation between the virulence of a CSFV strain and the amount of IFN produced during CSFV infection in swine has also been reported [22,29]. C-strain vaccinated pigs challenged at 6 days post-vaccination had a significant higher number of INF-γ secreting cells compared to mock vaccinated animals [18]. In a separate report it has been shown that protection at 5 days post-vaccination in C-strain vaccinated pigs was correlated with the presence of virus-specific INF-γ secreting cells that were detected as early as three days post-vaccination [16,17]. That report also demonstrated the direct effect of INF-γ on CSFV replication in cell cultures. In addition, the direct role of IFN-α in protection against challenge with a virulent CSFV strain has also been reported [30]. The findings from this study suggest that increased levels of IFN-α after FlagT4Gv vaccination may play a protective role against BICv challenge. Establishment of a robust antiviral state seems to be crucial to preventing virus replication and spread of challenge virus in animals vaccinated with LAVs.
